# 6β,8β-Dihy­droxy­eremophil-7(11)-en-8α,12-olide

**DOI:** 10.1107/S160053681104150X

**Published:** 2011-10-12

**Authors:** Zhan-Xin Zhang, Dong-Qing Fei

**Affiliations:** a School of Pharmacy, Lanzhou University, Lanzhou 730000, People’s Republic of China

## Abstract

The title compound, C_15_H_22_O_4_, an eremophilane sesquiternoid, was isolated from the roots of *Ligularia virgaurea*. Both six-membered rings (*A* and *B*) adopt chair conformations and the five-membered ring is almost planar (r.m.s. deviation = 0.016 Å). The two methyl and two hy­droxy groups adopt a *syn* conformation and the *A*/*B* ring junction is *cis*-fused. An intra­molecular O—H⋯O hydrogen bond generates an *S*(6) ring. In the crystal, O—H⋯O hydrogen bonds link the mol­ecules into [100] chains.

## Related literature

For further information on the isolation of the title compound, see Moriyama & Takahashi (1976 ▶); Zhang *et al.* (2008 ▶).
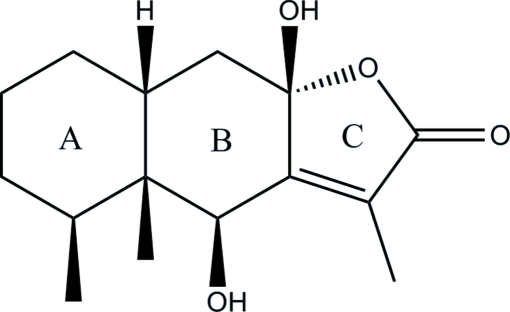


## Experimental

### 

#### Crystal data

C_15_H_22_O_4_
*M* *_r_* = 266.33
Orthorhombic, 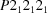

*a* = 9.8627 (7) Å
*b* = 10.5674 (7) Å
*c* = 13.1565 (9) Å
*V* = 1371.21 (16) Å^3^
*Z* = 4
Mo *K*α radiation
μ = 0.09 mm^−1^
*T* = 296 K
0.38 × 0.33 × 0.29 mm

#### Data collection

Bruker APEXII CCD diffractometer
Absorption correction: multi-scan (*SADABS*; Bruker, 2005 ▶) *T*_min_ = 0.966, *T*_max_ = 0.974
7478 measured reflections
2680 independent reflections
2306 reflections with *I* > 2σ(*I*)
*R*_int_ = 0.028

#### Refinement

*R*[*F* ^2^ > 2σ(*F* ^2^)] = 0.034
*wR*(*F* ^2^) = 0.087
*S* = 1.06
2680 reflections
178 parameters
H-atom parameters constrained
Δρ_max_ = 0.15 e Å^−3^
Δρ_min_ = −0.12 e Å^−3^

### 

Data collection: *APEX2* (Bruker, 2005 ▶); cell refinement: *SAINT* (Bruker, 2005 ▶); data reduction: *SAINT*; program(s) used to solve structure: *SHELXS97* (Sheldrick, 2008 ▶); program(s) used to refine structure: *SHELXL97* (Sheldrick, 2008 ▶); molecular graphics: *SHELXTL* (Sheldrick, 2008 ▶); software used to prepare material for publication: *SHELXTL*.

## Supplementary Material

Crystal structure: contains datablock(s) I, global. DOI: 10.1107/S160053681104150X/hb6428sup1.cif

Structure factors: contains datablock(s) I. DOI: 10.1107/S160053681104150X/hb6428Isup2.hkl

Additional supplementary materials:  crystallographic information; 3D view; checkCIF report

## Figures and Tables

**Table 1:** Hydrogen-bond geometry (Å, °)

*D*—H⋯*A*	*D*—H	H⋯*A*	*D*⋯*A*	*D*—H⋯*A*
O4—H4*A*⋯O3	0.82	2.11	2.8061 (19)	142
O3—H3⋯O1^i^	0.82	1.93	2.7502 (18)	174

Symmetry code: (i) 
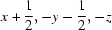
.

**Fig. 1.**
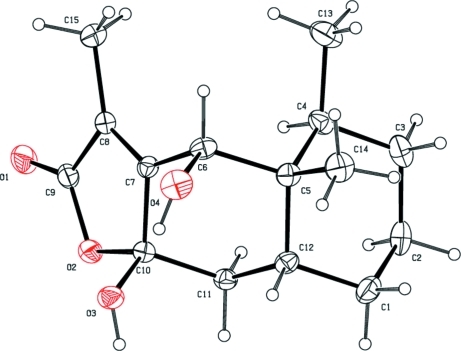
The molecular structure of the title compound showing 30% probability displacement ellipsoids.

## supplementary crystallographic information

### Comment

The title compound, 6*β*,8*β*-dihydroxyeremophil-7(11)-en-8*α*,12-olide (Fig. 1), was originally isolated from the *Ligularia fauriei* (Moriyama *et al.*, 1976). With the present compound, which was isolated from the roots of *L. virgaurea* (Zhang *et al.*, 2008). The title compound is composed of three rings, two six-membered and one five-membered. The two six-membered rings *A* and *B* adopt chair conformations with pucking paramaters Q = 0.559 (2) Å, θ = 176.0 (2)*^o^*, φ = 101 (3)° and Q = 0.5527 (18) Å, θ = 1.20 (19)*^o^*, φ = 127 (9)°, respectively. The five-membered ring *C* is almost planar with a mean torsion angle of 1.65 (8)°. The *A*/*B* ring junction is *cis*- fused. In the crystal, O—H···O hydrogen bonds occur.

### Experimental

The air-dried roots of *L. virgaurea* (3.8 kg) were pulverized and extracted with petroleum ether (60–90°C)—Et_2_O-MeOH (1: 1: 1) (6 days × 3 times) at room temperature. The extract was concentrated under reduced pressure giving a residue (256 g), which was chromatographed on a silica gel column (200–300 mesh) with a gradient of PE-acetone (AC) (30: 1; 15: 1; 8: 1; 5: 1; 3: 1; 1: 1 and 0: 1). According to TLC analysis, seven crude fractions (Fr. A—Fr. G) were collected. Fr. F was further fractionated on a silica gel column to obtain a mixture of the title compound and other compounds, which were purified by preparative TLC using PE—AC (2: 1) to give pure the title compound. Colourless blocks of the title compound were obtained after slow evaporation of a methanolic solution at room temperature.

### Refinement

The absolute structure was indeterminate in the present experiment. All H atoms were placed in geometrically calculated positions, and allowed to ride on their parent atoms with O—H = 0.82 Å and C—H = 0.96 - 0.98 Å, and with *U*_iso_(H) = 1.2Ueq(C) for methylene- and methine-H, and 1.5Ueq for other H atoms.

### Crystal data

**Table:**

C_15_H_22_O_4_	*D*_x_ = 1.290 Mg m^−^^3^
*M**_r_* = 266.33	Mo *K*α radiation, λ = 0.71073 Å
Orthorhombic, *P*2_1_2_1_2_1_	Cell parameters from 2527 reflections
*a* = 9.8627 (7) Å	θ = 2.5–24.7°
*b* = 10.5674 (7) Å	µ = 0.09 mm^−^^1^
*c* = 13.1565 (9) Å	*T* = 296 K
*V* = 1371.21 (16) Å^3^	Block, colourless
*Z* = 4	0.38 × 0.33 × 0.29 mm
*F*(000) = 576	

### Data collection

**Table:**

Bruker APEXII CCD diffractometer	2680 independent reflections
Radiation source: fine-focus sealed tube	2306 reflections with *I* > 2σ(*I*)
graphite	*R*_int_ = 0.028
φ and ω scans	θ_max_ = 26.0°, θ_min_ = 2.5°
Absorption correction: multi-scan (*SADABS*; Bruker, 2005)	*h* = −12→6
*T*_min_ = 0.966, *T*_max_ = 0.974	*k* = −13→12
7478 measured reflections	*l* = −16→15

### Refinement

**Table:**

Refinement on *F*^2^	Secondary atom site location: difference Fourier map
Least-squares matrix: full	Hydrogen site location: inferred from neighbouring sites
*R*[*F*^2^ > 2σ(*F*^2^)] = 0.034	H-atom parameters constrained
*wR*(*F*^2^) = 0.087	*w* = 1/[σ^2^(*F*_o_^2^) + (0.043*P*)^2^ + 0.0843*P*] where *P* = (*F*_o_^2^ + 2*F*_c_^2^)/3
*S* = 1.06	(Δ/σ)_max_ < 0.001
2680 reflections	Δρ_max_ = 0.15 e Å^−^^3^
178 parameters	Δρ_min_ = −0.12 e Å^−^^3^
0 restraints	Extinction correction: *SHELXTL* (Sheldrick, 2008), Fc^*^=kFc[1+0.001xFc^2^λ^3^/sin(2θ)]^-1/4^
Primary atom site location: structure-invariant direct methods	Extinction coefficient: 0.043 (3)

### Special details

**Table:**

Geometry. All e.s.d.'s (except the e.s.d. in the dihedral angle between two l.s. planes) are estimated using the full covariance matrix. The cell e.s.d.'s are taken into account individually in the estimation of e.s.d.'s in distances, angles and torsion angles; correlations between e.s.d.'s in cell parameters are only used when they are defined by crystal symmetry. An approximate (isotropic) treatment of cell e.s.d.'s is used for estimating e.s.d.'s involving l.s. planes.
Refinement. Refinement of *F*^2^ against ALL reflections. The weighted *R*-factor *wR* and goodness of fit *S* are based on *F*^2^, conventional *R*-factors *R* are based on *F*, with *F* set to zero for negative *F*^2^. The threshold expression of *F*^2^ > σ(*F*^2^) is used only for calculating *R*-factors(gt) *etc*. and is not relevant to the choice of reflections for refinement. *R*-factors based on *F*^2^ are statistically about twice as large as those based on *F*, and *R*- factors based on ALL data will be even larger.

### Fractional atomic coordinates and isotropic or equivalent isotropic displacement parameters (Å^2^)

**Table:**

	*x*	*y*	*z*	*U*_iso_*/*U*_eq_	
C1	0.52336 (19)	0.0280 (2)	0.02370 (15)	0.0528 (5)	
H1A	0.6033	0.0428	0.0650	0.063*	
H1B	0.5488	−0.0277	−0.0317	0.063*	
C2	0.4742 (2)	0.1530 (2)	−0.01946 (17)	0.0589 (6)	
H2A	0.4024	0.1371	−0.0682	0.071*	
H2B	0.5482	0.1941	−0.0550	0.071*	
C3	0.4214 (2)	0.24061 (19)	0.06327 (16)	0.0573 (5)	
H3A	0.3821	0.3150	0.0318	0.069*	
H3B	0.4970	0.2682	0.1049	0.069*	
C4	0.31514 (18)	0.17838 (17)	0.13139 (14)	0.0432 (4)	
H4	0.2371	0.1584	0.0881	0.052*	
C5	0.36613 (16)	0.05137 (17)	0.17644 (13)	0.0380 (4)	
C6	0.24978 (17)	−0.01496 (17)	0.23600 (12)	0.0401 (4)	
H6	0.2093	0.0468	0.2825	0.048*	
C7	0.14293 (16)	−0.05820 (15)	0.16381 (12)	0.0335 (4)	
C8	0.01487 (16)	−0.02563 (16)	0.14955 (12)	0.0360 (4)	
C9	−0.03401 (18)	−0.09426 (16)	0.05925 (13)	0.0405 (4)	
C10	0.18821 (16)	−0.14622 (15)	0.08141 (13)	0.0349 (4)	
C11	0.29893 (16)	−0.08367 (15)	0.01959 (12)	0.0359 (4)	
H11A	0.2612	−0.0128	−0.0178	0.043*	
H11B	0.3347	−0.1438	−0.0292	0.043*	
C12	0.41454 (16)	−0.03650 (17)	0.08853 (13)	0.0373 (4)	
H12	0.4563	−0.1111	0.1197	0.045*	
C13	0.2660 (3)	0.2734 (2)	0.21109 (18)	0.0673 (6)	
H13A	0.1857	0.2415	0.2434	0.101*	
H13B	0.2459	0.3527	0.1787	0.101*	
H13C	0.3355	0.2858	0.2612	0.101*	
C14	0.4830 (2)	0.0732 (2)	0.25188 (15)	0.0559 (5)	
H14A	0.5526	0.1226	0.2198	0.084*	
H14B	0.5198	−0.0069	0.2725	0.084*	
H14C	0.4495	0.1175	0.3104	0.084*	
C15	−0.07474 (19)	0.06430 (19)	0.20584 (15)	0.0498 (5)	
H15A	−0.0367	0.0811	0.2716	0.075*	
H15B	−0.1632	0.0276	0.2137	0.075*	
H15C	−0.0819	0.1420	0.1684	0.075*	
O1	−0.14548 (13)	−0.09203 (13)	0.02165 (11)	0.0576 (4)	
O2	0.06687 (12)	−0.16515 (11)	0.01970 (9)	0.0429 (3)	
O3	0.22670 (13)	−0.26232 (11)	0.12267 (9)	0.0471 (3)	
H3	0.2694	−0.3028	0.0802	0.071*	
O4	0.29776 (15)	−0.11964 (14)	0.29448 (10)	0.0555 (4)	
H4A	0.3033	−0.1824	0.2580	0.083*	

### Atomic displacement parameters (Å^2^)

**Table:**

	*U*^11^	*U*^22^	*U*^33^	*U*^12^	*U*^13^	*U*^23^
C1	0.0351 (9)	0.0668 (13)	0.0565 (11)	−0.0029 (9)	0.0062 (9)	−0.0042 (11)
C2	0.0502 (11)	0.0631 (13)	0.0635 (13)	−0.0217 (10)	0.0032 (11)	0.0153 (11)
C3	0.0551 (12)	0.0445 (11)	0.0723 (13)	−0.0125 (9)	−0.0128 (11)	0.0068 (10)
C4	0.0401 (10)	0.0368 (9)	0.0526 (10)	0.0005 (7)	−0.0124 (8)	−0.0072 (8)
C5	0.0328 (9)	0.0441 (10)	0.0369 (9)	0.0036 (8)	−0.0076 (7)	−0.0035 (8)
C6	0.0416 (10)	0.0486 (10)	0.0299 (8)	0.0076 (8)	−0.0010 (7)	−0.0035 (8)
C7	0.0347 (9)	0.0335 (9)	0.0322 (8)	−0.0004 (7)	0.0052 (7)	0.0024 (7)
C8	0.0349 (9)	0.0327 (9)	0.0406 (9)	−0.0038 (7)	0.0043 (8)	−0.0021 (7)
C9	0.0377 (9)	0.0339 (9)	0.0498 (10)	−0.0049 (8)	−0.0017 (8)	−0.0012 (8)
C10	0.0362 (9)	0.0331 (8)	0.0355 (8)	0.0005 (7)	0.0015 (7)	−0.0024 (7)
C11	0.0406 (9)	0.0345 (8)	0.0326 (8)	0.0018 (7)	0.0023 (7)	−0.0027 (7)
C12	0.0320 (8)	0.0398 (9)	0.0400 (9)	0.0069 (7)	0.0001 (7)	0.0024 (8)
C13	0.0713 (15)	0.0482 (12)	0.0823 (16)	0.0058 (11)	−0.0124 (12)	−0.0203 (11)
C14	0.0453 (11)	0.0706 (14)	0.0519 (10)	0.0003 (10)	−0.0178 (9)	−0.0061 (11)
C15	0.0436 (10)	0.0480 (11)	0.0577 (11)	0.0047 (9)	0.0100 (9)	−0.0039 (9)
O1	0.0394 (7)	0.0547 (8)	0.0786 (9)	−0.0039 (6)	−0.0148 (7)	−0.0134 (8)
O2	0.0402 (7)	0.0426 (7)	0.0458 (7)	−0.0028 (5)	−0.0015 (6)	−0.0104 (6)
O3	0.0542 (8)	0.0359 (7)	0.0511 (7)	0.0070 (6)	0.0127 (6)	0.0047 (5)
O4	0.0637 (9)	0.0639 (9)	0.0390 (7)	0.0101 (7)	−0.0063 (6)	0.0115 (6)

### Geometric parameters (Å, °)

**Table:**

C1—C2	1.517 (3)	C8—C15	1.494 (2)
C1—C12	1.531 (2)	C9—O1	1.206 (2)
C1—H1A	0.9700	C9—O2	1.350 (2)
C1—H1B	0.9700	C10—O3	1.3943 (19)
C2—C3	1.521 (3)	C10—O2	1.460 (2)
C2—H2A	0.9700	C10—C11	1.514 (2)
C2—H2B	0.9700	C11—C12	1.540 (2)
C3—C4	1.528 (3)	C11—H11A	0.9700
C3—H3A	0.9700	C11—H11B	0.9700
C3—H3B	0.9700	C12—H12	0.9800
C4—C13	1.531 (3)	C13—H13A	0.9600
C4—C5	1.551 (2)	C13—H13B	0.9600
C4—H4	0.9800	C13—H13C	0.9600
C5—C14	1.538 (2)	C14—H14A	0.9600
C5—C12	1.558 (2)	C14—H14B	0.9600
C5—C6	1.556 (2)	C14—H14C	0.9600
C6—O4	1.428 (2)	C15—H15A	0.9600
C6—C7	1.490 (2)	C15—H15B	0.9600
C6—H6	0.9800	C15—H15C	0.9600
C7—C8	1.323 (2)	O3—H3	0.8200
C7—C10	1.497 (2)	O4—H4A	0.8200
C8—C9	1.473 (2)		
			
C2—C1—C12	111.83 (15)	O1—C9—O2	121.66 (16)
C2—C1—H1A	109.2	O1—C9—C8	128.29 (17)
C12—C1—H1A	109.2	O2—C9—C8	110.06 (14)
C2—C1—H1B	109.2	O3—C10—O2	108.61 (12)
C12—C1—H1B	109.2	O3—C10—C7	110.25 (13)
H1A—C1—H1B	107.9	O2—C10—C7	104.09 (12)
C1—C2—C3	111.80 (17)	O3—C10—C11	113.40 (14)
C1—C2—H2A	109.3	O2—C10—C11	110.63 (13)
C3—C2—H2A	109.3	C7—C10—C11	109.46 (13)
C1—C2—H2B	109.3	C10—C11—C12	111.04 (13)
C3—C2—H2B	109.3	C10—C11—H11A	109.4
H2A—C2—H2B	107.9	C12—C11—H11A	109.4
C2—C3—C4	113.11 (16)	C10—C11—H11B	109.4
C2—C3—H3A	109.0	C12—C11—H11B	109.4
C4—C3—H3A	109.0	H11A—C11—H11B	108.0
C2—C3—H3B	109.0	C1—C12—C11	109.57 (14)
C4—C3—H3B	109.0	C1—C12—C5	111.28 (15)
H3A—C3—H3B	107.8	C11—C12—C5	113.78 (13)
C3—C4—C13	109.68 (16)	C1—C12—H12	107.3
C3—C4—C5	111.97 (15)	C11—C12—H12	107.3
C13—C4—C5	114.13 (15)	C5—C12—H12	107.3
C3—C4—H4	106.9	C4—C13—H13A	109.5
C13—C4—H4	106.9	C4—C13—H13B	109.5
C5—C4—H4	106.9	H13A—C13—H13B	109.5
C14—C5—C4	111.07 (15)	C4—C13—H13C	109.5
C14—C5—C12	109.80 (14)	H13A—C13—H13C	109.5
C4—C5—C12	109.35 (14)	H13B—C13—H13C	109.5
C14—C5—C6	107.17 (14)	C5—C14—H14A	109.5
C4—C5—C6	110.06 (14)	C5—C14—H14B	109.5
C12—C5—C6	109.34 (14)	H14A—C14—H14B	109.5
O4—C6—C7	109.88 (14)	C5—C14—H14C	109.5
O4—C6—C5	112.07 (14)	H14A—C14—H14C	109.5
C7—C6—C5	109.79 (13)	H14B—C14—H14C	109.5
O4—C6—H6	108.3	C8—C15—H15A	109.5
C7—C6—H6	108.3	C8—C15—H15B	109.5
C5—C6—H6	108.3	H15A—C15—H15B	109.5
C8—C7—C6	133.31 (15)	C8—C15—H15C	109.5
C8—C7—C10	110.12 (14)	H15A—C15—H15C	109.5
C6—C7—C10	116.18 (14)	H15B—C15—H15C	109.5
C7—C8—C9	107.39 (15)	C9—O2—C10	108.29 (12)
C7—C8—C15	131.31 (15)	C10—O3—H3	109.5
C9—C8—C15	121.29 (15)	C6—O4—H4A	109.5
			
C12—C1—C2—C3	54.1 (2)	C7—C8—C9—O2	−1.19 (19)
C1—C2—C3—C4	−52.5 (2)	C15—C8—C9—O2	177.43 (15)
C2—C3—C4—C13	−179.04 (17)	C8—C7—C10—O3	−118.86 (15)
C2—C3—C4—C5	53.2 (2)	C6—C7—C10—O3	67.41 (18)
C3—C4—C5—C14	67.24 (19)	C8—C7—C10—O2	−2.55 (17)
C13—C4—C5—C14	−58.1 (2)	C6—C7—C10—O2	−176.28 (13)
C3—C4—C5—C12	−54.10 (18)	C8—C7—C10—C11	115.75 (15)
C13—C4—C5—C12	−179.45 (15)	C6—C7—C10—C11	−57.99 (18)
C3—C4—C5—C6	−174.23 (14)	O3—C10—C11—C12	−70.31 (17)
C13—C4—C5—C6	60.42 (19)	O2—C10—C11—C12	167.39 (12)
C14—C5—C6—O4	−48.17 (19)	C7—C10—C11—C12	53.25 (17)
C4—C5—C6—O4	−169.07 (14)	C2—C1—C12—C11	69.93 (19)
C12—C5—C6—O4	70.79 (16)	C2—C1—C12—C5	−56.8 (2)
C14—C5—C6—C7	−170.58 (14)	C10—C11—C12—C1	−179.08 (14)
C4—C5—C6—C7	68.52 (17)	C10—C11—C12—C5	−53.80 (18)
C12—C5—C6—C7	−51.62 (18)	C14—C5—C12—C1	−66.08 (19)
O4—C6—C7—C8	121.9 (2)	C4—C5—C12—C1	56.03 (18)
C5—C6—C7—C8	−114.4 (2)	C6—C5—C12—C1	176.60 (13)
O4—C6—C7—C10	−66.24 (18)	C14—C5—C12—C11	169.56 (15)
C5—C6—C7—C10	57.46 (19)	C4—C5—C12—C11	−68.34 (17)
C6—C7—C8—C9	174.56 (17)	C6—C5—C12—C11	52.23 (17)
C10—C7—C8—C9	2.30 (18)	O1—C9—O2—C10	179.36 (15)
C6—C7—C8—C15	−3.9 (3)	C8—C9—O2—C10	−0.46 (18)
C10—C7—C8—C15	−176.13 (17)	O3—C10—O2—C9	119.19 (14)
C7—C8—C9—O1	179.00 (18)	C7—C10—O2—C9	1.73 (16)
C15—C8—C9—O1	−2.4 (3)	C11—C10—O2—C9	−115.75 (14)

### Hydrogen-bond geometry (Å, °)

**Table:**

*D*—H···*A*	*D*—H	H···*A*	*D*···*A*	*D*—H···*A*
O4—H4A···O3	0.82	2.11	2.8061 (19)	142
O3—H3···O1^i^	0.82	1.93	2.7502 (18)	174

Symmetry codes: (i) *x*+1/2, −*y*−1/2, −*z*.

## References

[bb1] Bruker (2005). *APEX2*, *SAINT* and *SADABS* Bruker AXS Inc., Madison, Wisconsin, USA.

[bb3] Moriyama, Y. & Takahashi, T. (1976). *Chem. Pharm. Bull.* **24**, 360–362.

[bb4] Sheldrick, G. M. (2008). *Acta Cryst.* A**64**, 112–122. 10.1107/S0108767307043930 18156677

[bb5] Zhang, Z.-X., Fei, D.-Q. & Jia, Z.-J. (2008). *Helv. Chim. Acta*, **91**, 1045–1052.

